# Glomerular TRPC5 and Palladin immunohistochemical profiling in focal segmental glomerulosclerosis: tissue heterogeneity and exploratory clinical associations (PRIME-PATH I)

**DOI:** 10.3389/fmed.2026.1924319

**Published:** 2026-10-22

**Authors:** Zeynep Ural, Betül Ogüt, Ipek Isık Gönül, Galip Güz

**Affiliations:** 1 Department of Nephrology, Gazi University Faculty of Medicine, Ankara, Türkiye; 2 Department of Pathology, Gazi University Faculty of Medicine, Ankara, Türkiye

**Keywords:** actin cytoskeleton, focal segmental glomerulosclerosis, immunohistochemistry, Palladin, podocyte, TRPC5

## Abstract

**Introduction:**

Focal segmental glomerulosclerosis (FSGS) is a shared histopathologic pattern arising from biologically diverse podocyte injuries. Transient receptor potential canonical 5 (TRPC5) has been implicated in calcium–cytoskeletal signaling and entered clinical evaluation, whereas Palladin is an actin-associated scaffold involved in cytoskeletal organization. Their glomerular immunohistochemical patterns in human FSGS remain poorly characterized.

**Methods:**

In this single-center retrospective biopsy cohort, 182 adults with biopsy-diagnosed FSGS between 2010 and 2020 were screened; 103 with adequate archived tissue, at least two evaluable glomeruli, and at least 24 months of follow-up comprised the analytic cohort. TRPC5 was scored as 0 (nuclear-only/no reproducible capillary-granular staining), 1 (discontinuous capillary-granular staining), or 2 (continuous/diffuse capillary-granular staining); scores 1–2 were grouped as TRPC5-positive. Palladin showed capillary-granular immunoreactivity in all evaluable biopsies and was classified as discontinuous or diffuse. Twenty-four-month treatment response was based on 24-hour urinary protein excretion. Poor response was the primary clinical outcome; kidney failure (maintenance dialysis or kidney transplantation) was evaluated during follow-up.

**Results:**

TRPC5 immunoreactivity was present in 70/103 biopsies (68.0%) and absent in 33/103 (32.0%); Palladin staining was diffuse in 75/103 (72.8%) and discontinuous in 28/103 (27.2%). Poor response occurred in 54/103 (52.4%) and kidney failure in 33/103 (32.0%). TRPC5-positive patients more often had poor response (64.3% vs. 27.3%; *P* < 0.001) and kidney failure (42.9% vs. 9.1%; *P* < 0.001). In the primary adjusted model, TRPC5 positivity was associated with poor response (OR, 3.28; 95% CI, 1.13–9.52; *P* = 0.029) and kidney failure (OR, 5.51; 95% CI, 1.25–24.23; *P* = 0.024). Estimates were similar after adjustment for global glomerulosclerosis but attenuated and imprecise after IFTA adjustment (*n* = 70). Palladin distribution was not independently associated with either outcome and did not improve model fit.

**Conclusion:**

PRIME-PATH I identifies heterogeneous glomerular TRPC5 immunoreactivity as a tissue-level phenotype within morphologically defined FSGS, with Palladin providing complementary cytoskeletal staining-distribution information. These associations are hypothesis-generating, and independent prognostic value remains uncertain after accounting for chronic structural injury. The findings do not establish functional TRPC5 activation or treatment predictiveness but support prospective studies integrating etiologic classification, cell-specific localization, pathway activity, and therapeutic response.

## Introduction

Focal segmental glomerulosclerosis (FSGS) is increasingly recognized not as a single disease entity but as a shared histopathologic pattern of podocyte injury arising from biologically diverse mechanisms. Contemporary frameworks distinguish primary/immune-mediated, genetic, maladaptive or other secondary, and undetermined forms because these categories differ substantially in pathogenesis, treatment indication, likelihood of remission, and kidney prognosis (1–7). This heterogeneity represents a central challenge for precision nephrology: renal biopsy provides an essential morphologic diagnosis and assessment of chronic injury, but morphology alone does not define the molecular process operating in an individual patient (4, 7, 8). Podocyte integrity depends on coordinated interactions among the slit diaphragm, actin cytoskeleton, cell–matrix adhesions, and intracellular signaling networks (9). Recent identification of anti-nephrin and other slit-diaphragm–directed antibodies further illustrates how apparently similar podocytopathy patterns can encompass biologically distinct disease states that may ultimately require different therapeutic approaches (10–12). In parallel, advances in podocyte mechanobiology emphasize that calcium signaling, Rho-family GTPases, actin dynamics, focal adhesions, and mechanical adaptation operate as an integrated injury-response system rather than as isolated molecular pathways (13). These concepts provide a rationale for complementing conventional morphology with tissue-level molecular phenotyping.

TRPC5 is a Ca^2^⁺-permeable, nonselective transient receptor potential canonical channel that has been implicated in Rac1-associated cytoskeletal dynamics. Experimental studies have linked TRPC5-mediated calcium entry to Rac1 signaling, synaptopodin destabilization, actin remodeling, and albuminuric injury, and genetic or pharmacologic inhibition of TRPC5 has been protective in selected experimental models (14–16). However, the mechanistic literature is not uniform. TRPC5 overexpression or activation has not consistently produced or aggravated glomerular injury, and Rac1-mediated podocyte injury has also been demonstrated independently of TRPC5 (17, 18). An updated review of renal calcium signaling similarly emphasized that the presence and functional contribution of TRPC5 in podocytes remain unresolved across experimental systems (19). Accordingly, detection of TRPC5 immunoreactivity in human kidney tissue should not be equated with channel activation, calcium influx, Rac1 activation, pathway dependence, or causality.

The translational development of TRPC5 nevertheless highlights an important gap between experimental target biology and human renal pathology. Selective TRPC5 inhibition progressed to clinical evaluation in FSGS, treatment-resistant minimal change disease, and diabetic nephropathy, and the TRACTION-2 program incorporated urinary Rac1 as a candidate predictive/pharmacodynamic biomarker (20). In contrast, a comprehensive *Physiological Reviews* synthesis noted that, at the time of its assessment, no published human renal-biopsy data had demonstrated disease-related changes in TRPC5 abundance comparable with those reported for TRPC6 (21). Thus, an intermediate translational layer remained insufficiently characterized: whether TRPC5 is uniformly represented in diseased human glomerular tissue or instead exhibits patient-to-patient tissue heterogeneity potentially relevant to clinical phenotype.

Palladin provides a complementary cytoskeletal dimension rather than an equivalent therapeutic or prognostic target. Palladin is a dynamic actin-associated scaffold protein involved in actin-filament organization, stress-fiber architecture, focal-adhesion stability, and cellular morphology and has been demonstrated in podocytes (22, 23). Experimental Palladin deficiency disrupts actin organization and focal-adhesion integrity, alters podocyte morphology, reduces expression of structural proteins including nephrin, and increases susceptibility to podocyte injury; importantly, reduced podocyte Palladin staining has also been reported in human FSGS and diabetic kidney disease biopsies (23). Conversely, Palladin can be upregulated in other renal compartments during injury, including epithelial repair responses and fibroblast-associated kidney fibrosis, and interstitial Palladin has been associated with adverse outcomes in diabetic kidney disease (24–26). These compartment- and context-dependent observations argue against interpreting glomerular Palladin staining as a simple surrogate of total protein abundance, podocyte-specific expression, or prognosis. Rather, Palladin staining distribution may provide complementary information about the cytoskeletal context in which glomerular injury is observed.

Against this background, PRIME-PATH I was designed to characterize glomerular TRPC5 and Palladin immunohistochemical patterns in human FSGS biopsy tissue and to examine their exploratory clinicopathologic associations. We specifically evaluated interindividual heterogeneity in TRPC5 capillary-granular immunoreactivity, characterized Palladin according to its glomerular capillary-granular distribution, and assessed their relationships with 24-month treatment response and kidney failure during follow-up. Palladin was evaluated in parallel as a complementary cytoskeletal staining phenotype rather than as a prespecified co-equal prognostic marker. To our knowledge, this is the first full-length systematic immunohistochemical characterization of TRPC5 and Palladin across a human FSGS biopsy cohort, providing a human-tissue bridge between experimental channel biology and future biomarker-integrated studies.

## Materials and methods

### Study design and patient selection

This was a single-center, retrospective clinicopathologic cohort study conducted at Gazi University Faculty of Medicine using archived diagnostic kidney-biopsy material and retrospectively collected clinical data. Adults aged ≥18 years with a recorded biopsy diagnosis of focal segmental glomerulosclerosis (FSGS) between 2010 and 2020 were screened. Eligibility for immunohistochemical analysis required available formalin-fixed paraffin-embedded (FFPE) tissue, at least two evaluable glomeruli on the immunostained sections, and at least 24 months of follow-up. Of 182 screened patients, 103 met these criteria and comprised the analytic cohort (Figure 1).

**Figure 1:**
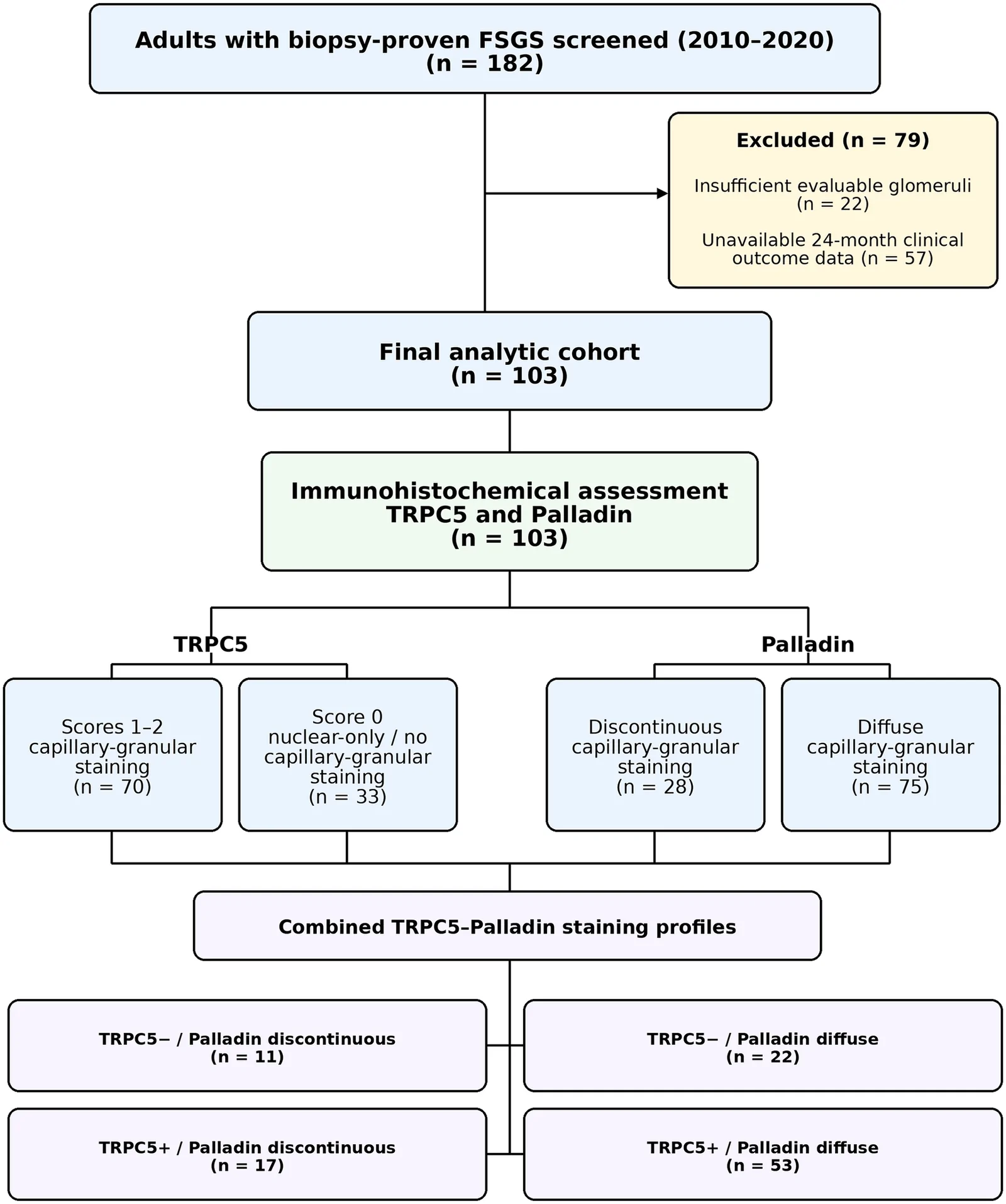
Study flow diagram. Among 182 screened adults with biopsy-proven FSGS (2010–2020), 103 comprised the final analytic cohort. Of the 79 excluded patients, 22 had insufficient evaluable glomeruli on the available immunostained sections and 57 had unavailable 24-month clinical outcome data. All included patients underwent TRPC5 and Palladin immunohistochemical assessment. TRPC5 score 0 denotes nuclear-only staining and/or absence of reproducible capillary-granular staining; scores 1–2 denote discontinuous or continuous/diffuse capillary-granular staining. Palladin was classified according to discontinuous versus diffuse capillary-granular distribution. FSGS, focal segmental glomerulosclerosis; IHC, immunohistochemistry; TRPC5, transient receptor potential canonical 5.

Of the 182 patients screened, 79 were excluded from the final analytic cohort: 22 because of insufficient evaluable glomerular tissue on the available immunostained sections and 57 because 24-month clinical outcome data were unavailable, primarily due to follow-up outside our institution.

The study was approved by the Gazi University Clinical Research Ethics Committee for Non-Interventional Studies (approval no. 406; April 19, 2021) and was conducted in accordance with the Declaration of Helsinki.

### FSGS classification and histopathologic evaluation

Histopathologic data were retrospectively abstracted from the original diagnostic kidney-biopsy reports. Recorded variables included total glomerular number, percentage of globally sclerotic glomeruli, presence of segmental sclerosis, interstitial fibrosis/tubular atrophy (IFTA), and the Columbia morphologic variant when specifically documented. IFTA was graded semiquantitatively as 0, 1, 2, or 3, corresponding to absent/minimal, mild, moderate, and severe chronic tubulointerstitial injury, respectively. IFTA grading was available in 70 patients (68.0%), whereas the percentage of globally sclerotic glomeruli was available for the entire analytic cohort.

A specific Columbia morphologic variant was documented in 74 of 103 biopsies: not otherwise specified (NOS), *n* = 53; tip lesion, *n* = 9; cellular, *n* = 5; perihilar, *n* = 3; and collapsing, *n* = 4. The remaining 29 biopsies were classified as unclassified/other because the archived pathology records did not support a specific variant assignment. Ninety-four biopsies were from native kidneys and nine from kidney allografts. FSGS was analyzed as a histopathologic diagnosis; retrospective etiologic subclassification was not performed because the clinical, genetic, and ultrastructural data required for reliable classification were not consistently available across the cohort.

### Clinical and laboratory data

Baseline was defined as the time of diagnostic kidney biopsy. Clinical variables included age, sex, hypertension, diabetes mellitus, serum creatinine, estimated glomerular filtration rate (eGFR), proteinuria, serum albumin, post-biopsy treatment exposure, 24-month treatment response, kidney-failure status, and total follow-up duration. eGFR was calculated using the Chronic Kidney Disease Epidemiology Collaboration creatinine equation used in the source dataset. Proteinuria at baseline and at 24 months was quantified by 24-hour urinary protein excretion.

Treatment exposure during the first 6 months after biopsy was categorized hierarchically as supportive therapy only, steroid-based therapy, or multi-agent/rescue immunosuppression. Supportive therapy included renin–angiotensin-system blockade and other non-immunosuppressive antiproteinuric or symptomatic care. Steroid-based therapy comprised corticosteroid treatment without an additional immunosuppressive agent. Multi-agent/rescue immunosuppression included calcineurin inhibitors, rituximab, mycophenolate mofetil, cyclophosphamide, or combination/sequential immunosuppressive regimens beyond corticosteroids alone. Any immunosuppressive exposure within the first 6 months was also recorded as present or absent.

### Outcomes

The primary clinical outcome was poor treatment response at 24 months. Response categories were determined using 24-hour urinary protein excretion in all included patients. Complete response was defined as post-treatment 24-hour urinary protein <0.5 g/day, partial response as a ≥ 50% reduction in 24-hour urinary protein from baseline, and no response as failure to meet either criterion. For the binary response outcome, complete and partial responses were grouped as responders, whereas no response was classified as poor response. Kidney failure was defined as initiation of maintenance dialysis or kidney transplantation during follow-up.

### Immunohistochemistry and staining interpretation

Four-micrometer sections were prepared from archived formalin-fixed paraffin-embedded kidney-biopsy blocks and mounted on positively charged glass slides. Immunohistochemical staining was performed on a BenchMark XT automated platform (Ventana Medical Systems/Roche, Tucson, AZ, USA). According to the archived laboratory protocol, staining was performed using an indirect immunoperoxidase procedure, with the protocol documenting a streptavidin–biotin three-step method and specifying the ultraView Universal DAB Detection Kit for chromogenic visualization. Antigen retrieval was performed in EDTA buffer (pH 8.0) for 1 h, followed by a 32-minute primary-antibody incubation. Sections were counterstained with Ventana hematoxylin, dehydrated through graded alcohols, cleared in xylene, and mounted with Entellan.

TRPC5 was detected using an affinity-purified rabbit polyclonal antibody raised against a synthetic peptide derived from human TRPC5 (Invitrogen/Thermo Fisher Scientific, catalog no. PA5-67556; RRID: AB_2688411; dilution 1:20), and Palladin using a rabbit polyclonal antibody (Proteintech, catalog no. 10853-1-AP; RRID: AB_2158782; dilution 1:200). The TRPC5 antibody was used at the supplier-tested dilution for human FFPE immunohistochemistry. Colonic mucosa and normal kidney tissue were used as positive-control tissues for TRPC5 and Palladin staining, respectively. Two renal pathologists, blinded to clinical outcome data, jointly evaluated the immunostained sections by light microscopy (Olympus BX51) and reached the final staining classification by consensus. A minimum of two evaluable glomeruli was required for staining assessment. Globally sclerotic glomeruli, purely extraglomerular signal, and nonreproducible background staining were excluded from the staining classification.

TRPC5 staining was classified according to the presence and distribution of reproducible glomerular capillary-granular immunoreactivity. Score 0 denoted nuclear-only staining and/or absence of reproducible capillary-granular staining, score 1 discontinuous capillary-granular staining, and score 2 continuous/diffuse capillary-granular staining. Scores 1 and 2 were grouped as TRPC5-positive for the primary binary analyses, whereas score 0 constituted the TRPC5-negative category.

Palladin showed glomerular capillary-granular immunoreactivity in all evaluable biopsies. Palladin score 0 denoted a discontinuous capillary-granular distribution, whereas score 1 denoted a diffuse capillary-granular distribution. Accordingly, Palladin was analyzed as a staining-distribution phenotype (discontinuous versus diffuse) rather than as negative versus positive expression. Representative staining patterns are shown in Figure 2.

**Figure 2:**
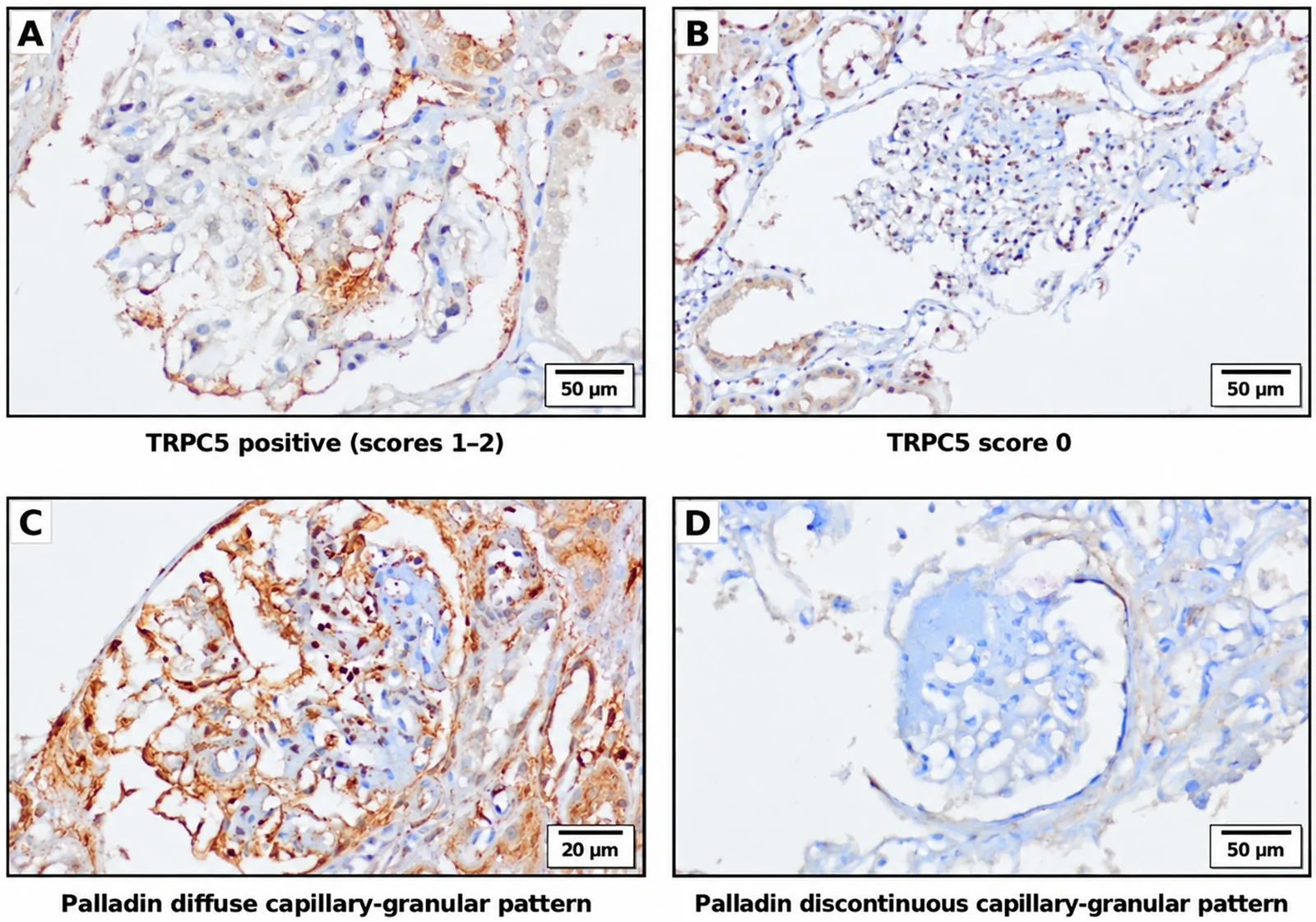
Representative glomerular TRPC5 and Palladin immunohistochemical staining patterns. **(A)** TRPC5 capillary-granular positive pattern (scores 1–2). **(B)** TRPC5 score 0 pattern, defined by nuclear-only staining and/or absence of reproducible glomerular capillary-granular signal. **(C)** Palladin diffuse capillary-granular pattern. **(D)** Palladin discontinuous capillary-granular pattern. At least two glomeruli were required for evaluability. The panels illustrate the operational glomerular staining categories used in this study; podocyte-specific colocalization was not performed. Original scale bars are shown within each panel.

### Statistical analysis

Continuous variables were summarized as median (interquartile range [IQR]) and categorical variables as number and percentage. Two-group comparisons of continuous variables were performed using the Mann–Whitney U test, whereas comparisons across the four TRPC5/Palladin staining profiles were performed using the Kruskal–Wallis test. Categorical variables were compared using the Pearson chi-square test or Fisher exact test, as appropriate. All statistical tests were two-sided.

For the primary analyses, TRPC5 was modeled as score 0 versus scores 1–2, and Palladin as discontinuous versus diffuse capillary-granular distribution. The four combined staining profiles, TRPC5-negative/Palladin-discontinuous, TRPC5-negative/Palladin-diffuse, TRPC5-positive/Palladin-discontinuous, and TRPC5-positive/Palladin-diffuse, were evaluated as exploratory categorical groups. Because these subgroup analyses were exploratory and some groups were small, overall omnibus tests were emphasized and no confirmatory pairwise comparisons were performed.

Multivariable logistic regression was used to evaluate associations with poor treatment response at 24 months and kidney failure during follow-up. The primary adjusted model included TRPC5 status, Palladin distribution, age, sex, baseline eGFR, and log2-transformed baseline 24-hour urinary protein excretion. Accordingly, the odds ratio for baseline proteinuria represents the change in odds associated with each doubling of proteinuria. TRPC5-negative and Palladin-discontinuous categories served as the reference groups.

A treatment-adjusted sensitivity model additionally included the three-level treatment category during the first 6 months after biopsy, with supportive therapy only as the reference category. Two additional sensitivity analyses assessed the robustness of the associations to adjustment for chronic structural kidney injury. In the IFTA complete-case analysis (*n* = 70), IFTA grade was added to the primary adjusted model and modeled ordinally per one-grade increase. In the full-cohort analysis (*n* = 103), the percentage of globally sclerotic glomeruli was added to the primary adjusted model and modeled per 10-percentage-point increase. Given the reduced sample size of the IFTA analysis and the limited number of kidney-failure events, estimates from these sensitivity models were interpreted with particular attention to their 95% confidence intervals.

The incremental contribution of Palladin distribution beyond TRPC5 and the clinical covariates was evaluated by comparing nested adjusted models with and without Palladin using likelihood-ratio tests, Akaike information criterion (AIC), and the apparent area under the receiver-operating-characteristic curve (AUC). A TRPC5×Palladin-distribution interaction term was additionally evaluated for poor treatment response. The corresponding interaction model for kidney failure was not stably estimable because no kidney-failure events occurred in the TRPC5-negative/Palladin-discontinuous subgroup, resulting in sparse-data separation. A native-kidney sensitivity analysis was performed after exclusion of the nine kidney-allograft biopsies (*n* = 94). Associations between TRPC5 status and poor response or kidney failure within this subset were evaluated using Fisher exact tests. Follow-up duration was compared between TRPC5-negative and TRPC5-positive groups using the Mann–Whitney U test. Kidney failure was analyzed as a binary outcome defined by the occurrence of maintenance dialysis or kidney transplantation during follow-up. Secondary sensitivity, interaction, incremental-value, and combined-profile analyses were considered exploratory, and no adjustment for multiple comparisons was applied. All statistical tests were two-sided, and *P* < 0.05 was considered statistically significant. Statistical analyses were performed using IBM SPSS Statistics for Windows, version 20.0 (IBM Corp., Armonk, NY, USA).

## Results

### Study cohort and baseline characteristics

Of 182 patients screened, 103 met the eligibility criteria and comprised the analytic cohort. The median age was 40 years (IQR, 28–50), and 55 patients (53.4%) were female. Median baseline eGFR was 68 mL/min/1.73 m^2^ (IQR, 60–90), median baseline 24-hour urinary protein excretion was 3986 mg/day (IQR, 2500–5847), and median serum albumin was 3.8 g/dL (IQR, 3.3–3.9). The median percentage of globally sclerotic glomeruli was 37% (IQR, 18–59), and IFTA grading was available in 70 patients (68.0%). Median follow-up was 48 months (IQR, 36–64). Ninety-four biopsies (91.3%) were from native kidneys and nine (8.7%) from kidney allografts. A specific Columbia morphologic variant was documented in 74 biopsies: not otherwise specified (NOS), *n* = 53; tip lesion, *n* = 9; cellular, *n* = 5; perihilar, *n* = 3; and collapsing, *n* = 4. The remaining 29 biopsies were classified as unclassified/other. During the first 6 months after biopsy, 33 patients (32.0%) received supportive therapy only, 60 (58.3%) steroid-based therapy, and 10 (9.7%) multi-agent/rescue immunosuppression (Table 1).

**Table 1:** Cohort characteristics, immunohistochemical profiles, and outcomes.

Characteristic	Overall cohort (*n* = 103)
Age, years	40 (28–50)
Female sex	55 (53.4)
Hypertension	36 (35.0)
Diabetes mellitus	9 (8.7)
Baseline eGFR, mL/min/1.73 m^2^	68 (60–90)
Baseline 24-hour urinary protein, mg/day	3,986 (2,500–5,847)
Serum albumin, g/dL	3.8 (3.3–3.9)
Globally sclerotic glomeruli, %	37 (18–59)
IFTA available	70 (68.0)
IFTA grade 0	12 (17.1)
IFTA grade 1	22 (31.4)
IFTA grade 2	26 (37.1)
IFTA grade 3	10 (14.3)
Native-kidney biopsy	94 (91.3)
Kidney allograft biopsy	9 (8.7)
Specific Columbia-type variant documented	74 (71.8)
NOS	53
Tip lesion	9
Cellular	5
Perihilar	3
Collapsing	4
Unclassified/other morphologic variant	29 (28.2)
Follow-up, months	48 (36–64)
Supportive therapy only	33 (32.0)
Steroid-based therapy	60 (58.3)
Multi-agent/rescue immunosuppression	10 (9.7)
Any immunosuppression within 6 months	64 (62.1)
TRPC5 positive (scores 1–2)	70 (68.0)
Palladin diffuse capillary-granular pattern	75 (72.8)
Palladin discontinuous capillary-granular pattern	28 (27.2)
TRPC5−/Palladin-discontinuous	11 (10.7)
TRPC5−/Palladin-diffuse	22 (21.4)
TRPC5+/Palladin-discontinuous	17 (16.5)
TRPC5+/Palladin-diffuse	53 (51.5)
Complete response at 24 months	30 (29.1)
Partial response at 24 months	19 (18.4)
No response at 24 months	54 (52.4)
Kidney failure during follow-up	33 (32.0)

Values are median (IQR) or *n* (%), unless otherwise indicated. Percentages for IFTA grades are calculated among patients with available IFTA data (*n* = 70). Columbia morphologic variants are based on specific labels recoverable from the archived pathology records; morphology does not establish FSGS etiology. TRPC5-positive denotes scores 1–2; TRPC5-negative denotes score 0. All included patients had at least 24 months of follow-up. For binary response analyses, complete and partial responses were grouped as responders (*n* = 49), whereas no response was classified as poor response (*n* = 54). eGFR, estimated glomerular filtration rate; FSGS, focal segmental glomerulosclerosis; IFTA, interstitial fibrosis and tubular atrophy; IQR, interquartile range; NOS, not otherwise specified; TRPC5, transient receptor potential canonical 5.

### TRPC5 and Palladin immunohistochemical profiles

Glomerular TRPC5 capillary-granular immunoreactivity was classified as positive in 70 patients (68.0%) and negative in 33 (32.0%). Palladin glomerular capillary-granular immunoreactivity was observed in all evaluable biopsies, with a diffuse distribution in 75 patients (72.8%) and a discontinuous distribution in 28 (27.2%). Accordingly, the four combined staining profiles comprised TRPC5-negative/Palladin-discontinuous in 11 patients (10.7%), TRPC5-negative/Palladin-diffuse in 22 (21.4%), TRPC5-positive/Palladin-discontinuous in 17 (16.5%), and TRPC5-positive/Palladin-diffuse in 53 (51.5%) (Table 1). Representative staining patterns are shown in Figure 2.

### Treatment response and kidney outcomes

At 24 months, 30 patients (29.1%) achieved complete response, 19 (18.4%) partial response, and 54 (52.4%) had no response. For binary analyses, complete and partial responses were grouped as responders (*n* = 49), whereas no response was classified as poor response (*n* = 54). Kidney failure occurred in 33 patients (32.0%) during follow-up. Compared with responders, poor responders had lower baseline eGFR and serum albumin, greater global glomerulosclerosis, and higher IFTA grade among patients with available IFTA data (Table 2). TRPC5 positivity was more frequent among poor responders than responders (83.3% vs. 51.0%; *P* < 0.001), whereas diffuse Palladin staining was not (75.9% vs. 69.4%; *P* = 0.456).

**Table 2:** Clinicopathologic characteristics according to 24-month treatment response.

Characteristic	Responders (*n* = 49)	Poor response (*n* = 54)	*P* value
Age, years	39 (26–53)	40 (29–49)	0.649
Female sex	26 (53.1)	29 (53.7)	0.948
Hypertension	12 (24.5)	24 (44.4)	0.034
Diabetes mellitus	4 (8.2)	5 (9.3)	1.000
Baseline eGFR, mL/min/1.73 m^2^	90 (82–90)	68 (56–68)	<0.001
Baseline 24-hour urinary protein, mg/day	3,000 (2,300–4,000)	4,000 (2,625–5,956)	0.132
Serum albumin, g/dL	3.9 (3.8–3.9)	3.8 (3.0–3.8)	<0.001
Globally sclerotic glomeruli, %	20 (11–38)	48 (36–61)	<0.001
IFTA grade	1 (0–2)	2 (1–2)	<0.001
Any immunosuppression within 6 months	19 (38.8)	45 (83.3)	<0.001
Treatment during the first 6 months after biopsy			<0.001
Supportive therapy only	25 (51.0)	8 (14.8)	
Steroid-based therapy	22 (44.9)	38 (70.4)	
Multi-agent/rescue immunosuppression	2 (4.1)	8 (14.8)	
TRPC5 positive (scores 1–2)	25 (51.0)	45 (83.3)	<0.001
Palladin diffuse capillary-granular pattern	34 (69.4)	41 (75.9)	0.456
Kidney failure during follow-up	3 (6.1)	30 (55.6)	<0.001

Values are median (IQR) or *n* (%), unless otherwise indicated. Responders comprise patients with complete or partial response at 24 months (*n* = 49); poor response denotes no response at 24 months (*n* = 54). IFTA comparisons are based on patients with available IFTA grading. *P* values are unadjusted and descriptive. Continuous variables were compared using the Mann–Whitney U test and categorical variables using Pearson's chi-square or Fisher's exact test, as appropriate. eGFR, estimated glomerular filtration rate; IFTA, interstitial fibrosis and tubular atrophy; IQR, interquartile range; TRPC5, transient receptor potential canonical 5.

Compared with TRPC5-negative patients, TRPC5-positive patients had lower baseline eGFR (*P* = 0.004), higher IFTA grade among patients with available IFTA data (*P* = 0.043), and more frequent immunosuppressive treatment within the first 6 months after biopsy (*P* = 0.005). Poor response occurred in 64.3% of TRPC5-positive versus 27.3% of TRPC5-negative patients (*P* < 0.001), and kidney failure occurred in 42.9% versus 9.1%, respectively (*P* < 0.001) (Table 3; Figure 3). Median follow-up duration was similar between TRPC5-negative and TRPC5-positive groups (44 [IQR, 36–72] vs. 48 [IQR, 37–60] months; *P* = 0.763). Diffuse versus discontinuous Palladin distribution was not significantly associated with poor response (54.7% vs. 46.4%; *P* = 0.456), kidney failure (34.7% vs. 25.0%; *P* = 0.350), IFTA grade (*P* = 0.205), or global glomerulosclerosis (*P* = 0.227) (Table 4). Complete response was less frequent in the diffuse-pattern group than in the discontinuous-pattern group (22.7% vs. 46.4%; *P* = 0.018). Across the four combined TRPC5/Palladin staining profiles, poor response differed across groups (overall *P* = 0.002) and occurred predominantly in the two TRPC5-positive groups; kidney failure showed a similar distribution (overall *P* = 0.006) (Table 5; Figure 4).

**Table 3:** Clinicopathologic characteristics and outcomes according to TRPC5 immunohistochemical status.

Characteristic	TRPC5 negative (*n* = 33)	TRPC5 positive (*n* = 70)	*P* value
Age, years	40 (29–52)	39 (28–50)	0.854
Female sex	15 (45.5)	40 (57.1)	0.267
Hypertension	12 (36.4)	24 (34.3)	0.836
Diabetes mellitus	1 (3.0)	8 (11.4)	0.265
Baseline eGFR, mL/min/1.73 m^2^	82 (82–90)	68 (56–90)	0.004
Baseline 24-hour urinary protein, mg/day	4,000 (2,700–6,000)	3,756 (2,222–5,575)	0.417
Serum albumin, g/dL	3.8 (3.8–3.9)	3.8 (3.3–3.9)	0.082
Globally sclerotic glomeruli, %	25 (20–42)	43 (18–60)	0.059
IFTA grade	1 (0–2)	2 (1–2)	0.043
Any immunosuppression within 6 months	14 (42.4)	50 (71.4)	0.005
Treatment during the first 6 months after biopsy			0.025
Supportive therapy only	16 (48.5)	17 (24.3)	
Steroid-based therapy	13 (39.4)	47 (67.1)	
Multi-agent/rescue immunosuppression	4 (12.1)	6 (8.6)	
Palladin diffuse capillary-granular pattern	22 (66.7)	53 (75.7)	0.336
Complete response at 24 months	22 (66.7)	8 (11.4)	<0.001
Poor response at 24 months	9 (27.3)	45 (64.3)	<0.001
Kidney failure during follow-up	3 (9.1)	30 (42.9)	<0.001
Follow-up, months	44 (36–72)	48 (37–60)	0.763

Values are median (IQR) or *n* (%), unless otherwise indicated. TRPC5-negative denotes score 0 (nuclear-only staining and/or absence of reproducible glomerular capillary-granular immunoreactivity), whereas TRPC5-positive denotes scores 1–2 (discontinuous or continuous/diffuse capillary-granular immunoreactivity). IFTA comparisons are based on patients with available IFTA grading. *P* values are unadjusted and descriptive. Continuous variables were compared using the Mann–Whitney U test and categorical variables using Pearson's chi-square or Fisher's exact test, as appropriate. eGFR, estimated glomerular filtration rate; IFTA, interstitial fibrosis and tubular atrophy; IQR, interquartile range; TRPC5, transient receptor potential canonical 5.

**Figure 3:**
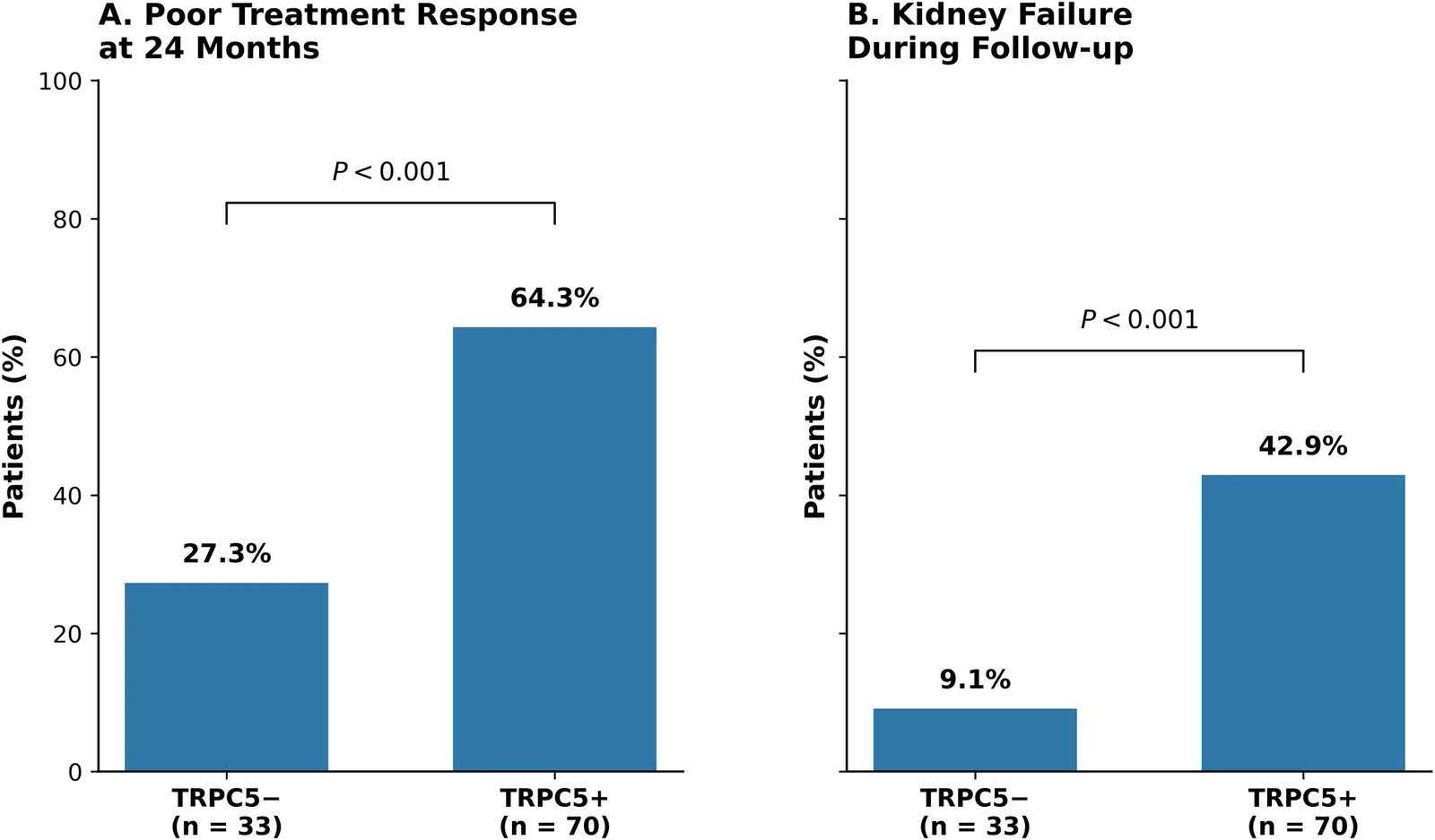
Clinical outcomes according to TRPC5 staining. Poor response at 24 months **(A)** and kidney failure during follow-up **(B)** were more frequent in TRPC5-positive than TRPC5-negative patients. TRPC5-negative denotes score 0, whereas TRPC5-positive denotes scores 1–2. Bars represent the percentage of patients in each group; group sizes are shown below the *x*-axis labels. *P* values are unadjusted between-group comparisons.

**Table 4:** Clinicopathologic characteristics and outcomes according to Palladin capillary-granular distribution.

Characteristic	Palladin discontinuous (*n* = 28)	Palladin diffuse (*n* = 75)	*P* value
Age, years	36 (29–50)	41 (28–50)	0.533
Female sex	11 (39.3)	44 (58.7)	0.079
Hypertension	11 (39.3)	25 (33.3)	0.573
Diabetes mellitus	2 (7.1)	7 (9.3)	1.000
Baseline eGFR, mL/min/1.73 m^2^	82 (59–90)	68 (63–90)	0.601
Baseline 24-hour urinary protein, mg/day	3,800 (2,700–5,982)	3,986 (2,300–5,700)	0.640
Serum albumin, g/dL	3.8 (3.2–3.9)	3.8 (3.3–3.9)	0.853
Globally sclerotic glomeruli, %	46 (20–66)	37 (16–52)	0.227
IFTA grade	1 (1–2)	2 (1–2)	0.205
Any immunosuppression within 6 months	16 (57.1)	48 (64.0)	0.523
Treatment during the first 6 months after biopsy			0.041
Supportive therapy only	12 (42.9)	21 (28.0)	
Steroid-based therapy	11 (39.3)	49 (65.3)	
Multi-agent/rescue immunosuppression	5 (17.9)	5 (6.7)	
TRPC5 positive (scores 1–2)	17 (60.7)	53 (70.7)	0.336
Complete response at 24 months	13 (46.4)	17 (22.7)	0.018
Poor response at 24 months	13 (46.4)	41 (54.7)	0.456
Kidney failure during follow-up	7 (25.0)	26 (34.7)	0.350

Values are median (IQR) or *n* (%), unless otherwise indicated. Palladin was classified according to glomerular capillary-granular staining distribution as discontinuous or diffuse; all evaluable biopsies showed glomerular Palladin immunoreactivity, and there was no Palladin-negative analytic group. IFTA comparisons are based on patients with available IFTA grading. *P* values are unadjusted and descriptive. Continuous variables were compared using the Mann–Whitney U test and categorical variables using Pearson's chi-square or Fisher's exact test, as appropriate. eGFR, estimated glomerular filtration rate; IFTA, interstitial fibrosis and tubular atrophy; IQR, interquartile range; TRPC5, transient receptor potential canonical 5.

**Table 5:** Clinicopathologic characteristics and outcomes across combined TRPC5–Palladin staining profiles.

Characteristic	TRPC5−/Palladin-discontinuous (*n* = 11)	TRPC5−/Palladin-diffuse (*n* = 22)	TRPC5+/Palladin-discontinuous (*n* = 17)	TRPC5+/Palladin-diffuse (*n* = 53)	*P* value
Age, years	36 (28–52)	44 (29–48)	36 (30–44)	41 (28–50)	0.926
Female sex	5 (45.5)	10 (45.5)	6 (35.3)	34 (64.2)	0.136
Baseline eGFR, mL/min/1.73 m^2^	90 (82–90)	82 (72–90)	68 (56–90)	68 (56–90)	0.026
Baseline 24-hour urinary protein, mg/day	2,700 (2,700–3,800)	4,000 (2,947–6,000)	4,400 (2,700–6,200)	3,500 (2,000–4,300)	0.133
Globally sclerotic glomeruli, %	20 (19–40)	27 (20–42)	60 (43–71)	37 (15–57)	0.055
IFTA grade	1 (0–2)	1 (0–2)	1 (1–2)	2 (1–2)	0.140
Any immunosuppression within 6 months	4 (36.4)	10 (45.5)	12 (70.6)	38 (71.7)	0.041
Complete response at 24 months	10 (90.9)	12 (54.5)	3 (17.6)	5 (9.4)	<0.001
Partial response at 24 months	0 (0.0)	2 (9.1)	2 (11.8)	15 (28.3)	0.053
Poor response at 24 months	1 (9.1)	8 (36.4)	12 (70.6)	33 (62.3)	0.002
Kidney failure during follow-up	0 (0.0)	3 (13.6)	7 (41.2)	23 (43.4)	0.006

Values are median (IQR) or *n* (%), unless otherwise indicated. Palladin categories represent discontinuous versus diffuse glomerular capillary-granular staining distribution and do not denote negative versus positive staining. IFTA comparisons are based on patients with available IFTA grading. Combined-profile analyses were descriptive and exploratory because of the small subgroup sizes. *P* values are overall comparisons across the four groups; no confirmatory pairwise comparisons were performed. Continuous variables were compared using the Kruskal–Wallis test and categorical variables using Pearson's chi-square or Fisher's exact test, as appropriate. eGFR, estimated glomerular filtration rate; IFTA, interstitial fibrosis and tubular atrophy; IQR, interquartile range; TRPC5, transient receptor potential canonical 5.

**Figure 4:**
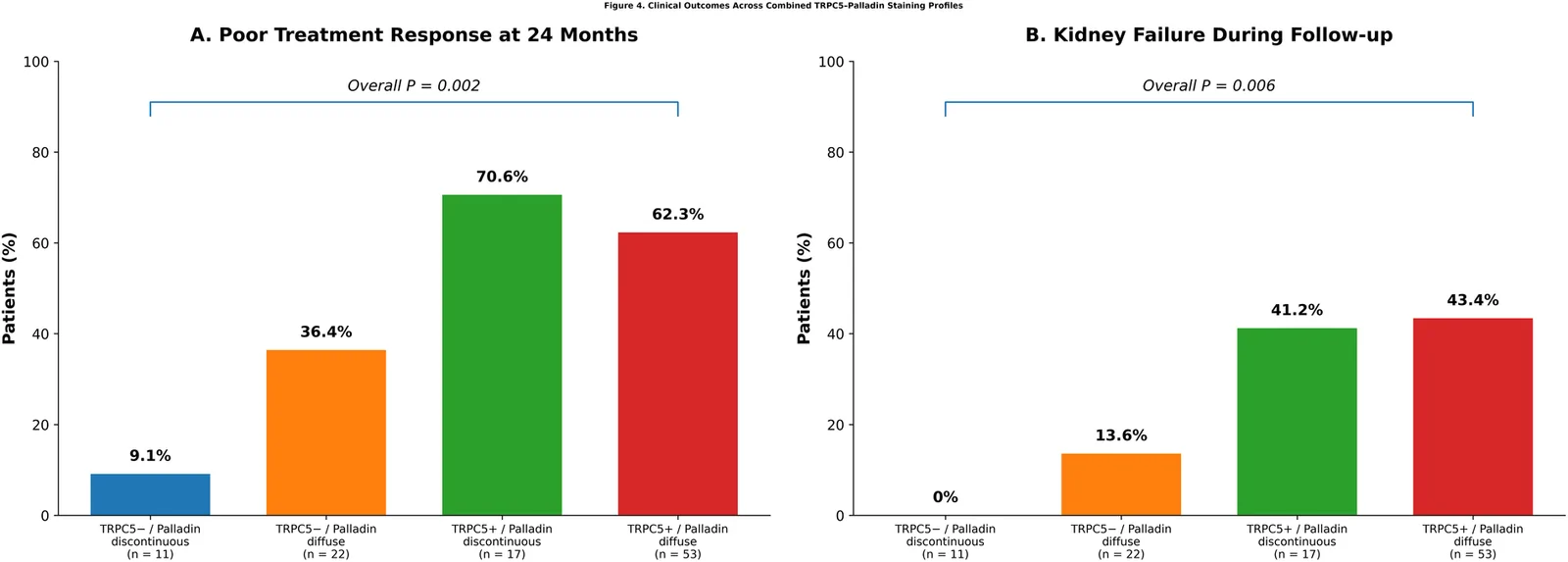
Clinical outcomes across combined TRPC5–Palladin staining profiles. Poor response at 24 months **(A)** and kidney failure during follow-up **(B)** are shown across the four combined TRPC5/Palladin staining profiles. Palladin categories represent discontinuous versus diffuse capillary-granular distribution, not negative versus positive staining. The four-group analyses were descriptive and exploratory. Overall *P* values compare all four groups; no confirmatory pairwise comparisons were performed. Bars represent the percentage of patients in each subgroup, with group sizes shown below the *x*-axis labels.

### Multivariable analyses

In the primary adjusted model, TRPC5 positivity was associated with poor response (OR, 3.28; 95% CI, 1.13–9.52; *P* = 0.029) and kidney failure (OR, 5.51; 95% CI, 1.25–24.23; *P* = 0.024). Diffuse Palladin distribution was not significantly associated with poor response (OR, 1.34; 95% CI, 0.42–4.32; *P* = 0.620) or kidney failure (OR, 2.46; 95% CI, 0.65–9.24; *P* = 0.183). After additional adjustment for treatment category during the first 6 months, TRPC5 positivity remained associated with poor response (OR, 3.26; 95% CI, 1.03–10.29; *P* = 0.044) and kidney failure (OR, 6.12; 95% CI, 1.15–32.43; *P* = 0.033) (Table 6).

**Table 6:** Multivariable logistic regression and chronicity sensitivity analyses.

Outcome	Model	Variable	n	OR (95% CI)	*P* value
Poor response	Primary adjusted	TRPC5 positive	103	3.28 (1.13–9.52)	0.029
		Palladin diffuse pattern	103	1.34 (0.42–4.32)	0.620
Poor response	Treatment-adjusted	TRPC5 positive	103	3.26 (1.03–10.29)	0.044
Poor response	IFTA complete-case	TRPC5 positive	70	2.27 (0.52–9.97)	0.279
		IFTA grade, per one-grade increase	70	2.73 (1.11–6.71)	0.029
Poor response	Global-sclerosis adjusted	TRPC5 positive	103	3.42 (1.15–10.20)	0.027
		Globally sclerotic glomeruli, per 10% increase	103	1.32 (1.03–1.68)	0.028
Kidney failure	Primary adjusted	TRPC5 positive	103	5.51 (1.25–24.23)	0.024
		Palladin diffuse pattern	103	2.46 (0.65–9.24)	0.183
Kidney failure	Treatment-adjusted	TRPC5 positive	103	6.12 (1.15–32.43)	0.033
Kidney failure	IFTA complete-case	TRPC5 positive	70	4.12 (0.55–31.12)	0.170
		IFTA grade, per one-grade increase	70	2.55 (0.90–7.22)	0.078
Kidney failure	Global-sclerosis adjusted	TRPC5 positive	103	5.54 (1.25–24.63)	0.024
		Globally sclerotic glomeruli, per 10% increase	103	1.12 (0.86–1.47)	0.392

ORs and 95% CIs are from multivariable logistic regression models. The primary adjusted model included TRPC5 status, Palladin distribution (diffuse vs. discontinuous), age, sex, baseline eGFR, and log2-transformed baseline 24-hour urinary protein excretion. The treatment-adjusted model additionally included treatment category during the first 6 months after biopsy. The IFTA complete-case sensitivity model added IFTA grade to the primary covariates in patients with available IFTA data (*n* = 70), whereas the global-sclerosis sensitivity model added the percentage of globally sclerotic glomeruli in the full cohort (*n* = 103). TRPC5-negative and Palladin-discontinuous categories served as reference groups. Only coefficients directly relevant to the study hypotheses and chronicity sensitivity analyses are presented. CI, confidence interval; eGFR, estimated glomerular filtration rate; IFTA, interstitial fibrosis and tubular atrophy; OR, odds ratio; TRPC5, transient receptor potential canonical 5.

In the IFTA complete-case sensitivity analysis (*n* = 70), the association of TRPC5 positivity with poor response was attenuated (OR, 2.27; 95% CI, 0.52–9.97; *P* = 0.279), whereas higher IFTA grade was associated with poor response (OR, 2.73 per one-grade increase; 95% CI, 1.11–6.71; *P* = 0.029). For kidney failure, the corresponding TRPC5 estimate was OR 4.12 (95% CI, 0.55–31.12; *P* = 0.170), and the OR for IFTA grade was 2.55 per one-grade increase (95% CI, 0.90–7.22; *P* = 0.078). In the full-cohort sensitivity analysis incorporating global glomerulosclerosis, TRPC5 positivity was associated with poor response (OR, 3.42; 95% CI, 1.15–10.20; *P* = 0.027) and kidney failure (OR, 5.54; 95% CI, 1.25–24.63; *P* = 0.024). Each 10-percentage-point increase in globally sclerotic glomeruli was associated with poor response (OR, 1.32; 95% CI, 1.03–1.68; *P* = 0.028), but not with kidney failure (OR, 1.12; 95% CI, 0.86–1.47; *P* = 0.392) (Table 6; Supplementary Table S1).

Adding Palladin distribution to the adjusted model did not improve model fit for poor response (likelihood-ratio *P* = 0.620; AIC, 112.32 without vs. 114.07 with Palladin; apparent AUC, 0.850 vs. 0.849). The TRPC5×Palladin-distribution interaction was not significant (OR, 0.28; 95% CI, 0.018–4.36; *P* = 0.364; nested likelihood-ratio *P* = 0.342). For kidney failure, addition of Palladin distribution likewise did not significantly improve model fit (likelihood-ratio *P* = 0.173; AIC, 100.32 vs. 100.46; apparent AUC, 0.853 vs. 0.861). The corresponding interaction model was not stably estimable because no kidney-failure events occurred in the TRPC5-negative/Palladin-discontinuous subgroup. In the native-kidney sensitivity analysis (*n* = 94), poor response occurred in 38 of 63 TRPC5-positive patients (60.3%) and 9 of 31 TRPC5-negative patients (29.0%; *P* = 0.008). Kidney failure occurred in 24 of 63 (38.1%) and 3 of 31 (9.7%), respectively (*P* = 0.004) (Supplementary Table S1).

## Discussion

PRIME-PATH I characterizes a previously underexplored translational layer in FSGS: the tissue-level representation of TRPC5 in diseased human glomeruli. To our knowledge, this is the first full-length systematic immunohistochemical characterization of TRPC5 across a human FSGS biopsy cohort. Glomerular TRPC5 capillary-granular immunoreactivity showed marked interindividual heterogeneity, being absent in approximately one third of biopsies and present in two thirds. This observation addresses a specific gap highlighted in a major review of glomerular ion-channel biology, which noted the absence of published human renal-biopsy data demonstrating disease-associated changes in TRPC5 abundance comparable with those described for TRPC6 (21). The principal contribution of the present study is therefore the demonstration of a heterogeneous TRPC5 tissue phenotype within morphologically defined FSGS, rather than validation of TRPC5 as a prognostic biomarker.

TRPC5-positive biopsies were enriched among patients with poor 24-month treatment response and kidney failure during follow-up. These associations remained evident in the primary clinical model, after adjustment for treatment category, and in the full-cohort model incorporating global glomerulosclerosis. Proteinuria remission is strongly associated with kidney survival in FSGS (27, 28), making the observed separation in treatment response clinically relevant. However, the chronicity analyses substantially qualify this finding. TRPC5-positive patients had lower baseline eGFR and greater IFTA, and poor responders had markedly greater global glomerulosclerosis and IFTA. In the IFTA complete-case model, the TRPC5 estimates were attenuated and imprecise, whereas IFTA grade was associated with poor response. By contrast, TRPC5 remained associated with both outcomes after adjustment for global glomerulosclerosis in the full cohort. These complementary analyses do not establish that TRPC5 provides prognostic information independent of chronic structural injury. Rather, TRPC5 immunoreactivity may identify an injury phenotype that accompanies disease severity, becomes enriched as chronic damage evolves, or reflects biological processes that are only partly captured by conventional chronicity measures.

This distinction is particularly important because FSGS is a shared histopathologic lesion rather than a single molecular disease (2–7). Similar segmental sclerotic lesions can arise from immune-mediated, genetic, maladaptive, toxic, and other podocyte injuries, with different implications for therapy and prognosis. Contemporary precision-nephrology frameworks therefore seek to integrate clinical phenotype, morphology, genetics, and molecular information rather than infer etiology from morphology alone (4, 7, 8). Recent work on anti-nephrin and other slit-diaphragm antibodies illustrates this principle: molecularly defined immune-mediated podocytopathy can occur within conventional minimal-change disease or FSGS morphologic patterns, and tissue localization can contribute information not apparent from light-microscopic classification alone (10–12). The present findings extend this concept in a different direction by demonstrating marked interindividual heterogeneity in glomerular TRPC5-directed immunoreactivity within FSGS biopsies. Importantly, the current data do not establish that these staining categories correspond to distinct etiologies or functional pathway states.

The biological rationale for studying TRPC5 is substantial but remains contested. Experimental studies have linked TRPC5-dependent calcium entry to Rac1-associated cytoskeletal remodeling and have shown protection from proteinuric injury with genetic or pharmacologic TRPC5 inhibition in selected models (14–16). Conversely, other studies have not reproduced a disease-promoting effect of TRPC5 overexpression or activation, and Rac1-driven podocyte injury has been demonstrated independently of TRPC5 (17, 18). Contemporary reviews therefore continue to regard the abundance, activation, and functional relevance of podocyte TRPC5 as incompletely resolved across experimental systems (19, 21). This uncertainty is consistent with a broader mechanobiological model in which podocyte behavior emerges from integrated calcium signaling, Rho-family GTPases, actin dynamics, cell–matrix adhesion, and mechanical forces rather than from a single ion channel in isolation (13). Accordingly, the immunohistochemical phenotype observed here should not be interpreted as evidence of increased TRPC5 channel activity, calcium influx, Rac1 activation, or pathway dependence. Nevertheless, renal and podocyte-associated TRPC5 localization has been supported by orthogonal experimental approaches. Schaldecker et al. demonstrated loss of renal TRPC5 immunoreactivity in Trpc5-knockout tissue together with podocin and synaptopodin colocalization, whereas Wang et al. supported renal TRPC5 expression using qPCR, Western blotting with independent monoclonal-antibody confirmation, and immunofluorescent colocalization with synaptopodin (15, 17). These studies provide independent biological support for podocyte-associated TRPC5 in experimental kidney systems but do not specifically validate PA5-67556 in human FFPE kidney tissue.

A related limitation concerns cellular localization. TRPC5 classification in this study was based on reproducible glomerular capillary-granular immunoreactivity, but double labeling or serial-section colocalization with nephrin, podocin, synaptopodin, or WT1 was not performed. The signal therefore cannot be assigned definitively to podocytes. This distinction is increasingly important as renal pathology moves toward spatially resolved molecular classification: recent podocytopathy studies have combined biopsy staining with cell-specific markers and high-resolution imaging to define the cellular compartment of molecular abnormalities (10–12). Future studies of TRPC5 should similarly combine quantitative tissue assessment with podocyte-specific colocalization and orthogonal measures of target abundance and pathway activity.

Palladin provides complementary information on glomerular cytoskeletal organization, but the present data do not support its use as a co-equal prognostic marker. Palladin is an actin-associated scaffold involved in cytoskeletal organization, focal adhesions, and cellular remodeling (22, 23). Experimental Palladin deficiency disrupts podocyte architecture and is associated with reduced nephrin and focal-adhesion integrity, while a small human biopsy series reported reduced podocyte Palladin in FSGS and diabetic kidney disease (23). At the same time, Palladin biology is strongly compartment dependent. Palladin can be upregulated in injured renal compartments (24), and recent work has identified fibroblast Palladin as a mediator of kidney fibrosis through actin-dependent MRTF–SRF signaling (25). In public human FSGS datasets analyzed in that study, tubulointerstitial PALLD expression correlated inversely with GFR and positively with COL1A1 and IFTA, further linking interstitial Palladin to chronic structural injury (25). Consistent with this compartment-specific profibrotic phenotype, a 2026 cohort of patients with biopsy-proven diabetic kidney disease showed that greater interstitial Palladin expression was associated with lower baseline eGFR and poorer event-free survival; each doubling of Palladin-positive area was associated with a higher age-adjusted risk of kidney replacement therapy or death (26). These observations reinforce the compartment-dependent biology of Palladin and caution against interpreting the glomerular diffuse/discontinuous staining phenotype observed in our cohort as a simple surrogate of total Palladin abundance or prognostic effect. In the present cohort, all evaluable biopsies showed glomerular Palladin immunoreactivity, with heterogeneity expressed as discontinuous versus diffuse capillary-granular distribution. Diffuse Palladin distribution was associated with a lower unadjusted complete-response frequency but was not associated with the binary poor-response endpoint, kidney failure, IFTA grade, global glomerulosclerosis, or improved adjusted model performance. Palladin is therefore best interpreted here as a complementary glomerular cytoskeletal staining-distribution phenotype rather than as an independently validated prognostic biomarker.

The translational relevance of this distinction is underscored by the evolving therapeutic landscape of podocytopathies. Selective TRPC5 inhibition entered clinical evaluation in TRACTION-2, which incorporated urinary Rac1 as a candidate pharmacodynamic and enrichment biomarker (20), but pretreatment renal-biopsy TRPC5 phenotype was not established as a treatment-selection marker. TRPC6 provides a useful, although biologically distinct, comparator. Genetic and acquired-disease studies established TRPC6 as a human podocytopathy pathway (29–33), and a recent randomized phase 2 trial reported reduction in proteinuria with the selective TRPC6 inhibitor BI 764198 in FSGS (34). More broadly, the emerging precision-medicine framework in FSGS argues for moving beyond morphology alone by integrating etiologic, genetic, biomarker, and molecular information to resolve biologically distinct disease subsets (35). This concept is particularly relevant across podocytopathies, in which a shared pattern of podocyte injury may arise from fundamentally different molecular disturbances. Work in Alport syndrome illustrates the broader principle that podocyte-directed therapeutic strategies can be developed around defined mechanisms of cellular injury beyond primary FSGS (36), while recent FSGS literature highlights an expanding spectrum of pathway-oriented approaches targeting cytoskeletal dysregulation, oxidative stress, mitochondrial dysfunction, autophagy, immune signaling, and biomarker-defined mechanisms (37). Viewed through this precision-pathology framework, tissue molecular phenotyping may provide a complementary layer of information by identifying heterogeneity in tissue-level immunohistochemical phenotypes within diseased glomeruli and thereby helping to bridge conventional histopathology with mechanistic disease classification. These developments support the broader feasibility of pathway-directed podocyte therapy but do not establish therapeutic relevance or treatment predictiveness of TRPC5 immunoreactivity. Rather, they highlight an important translational principle: tissue target abundance or distribution, functional pathway activity, disease etiology, and therapeutic response represent distinct but potentially complementary biological layers that should be measured and linked prospectively rather than inferred from one another.

Taken together, the findings support a precision-pathology framework in which an FSGS biopsy can potentially provide information beyond the presence and chronicity of segmental sclerosis. The relevant question is not whether TRPC5 immunoreactivity defines a new etiologic subtype of FSGS, it does not on the basis of the present data, but whether tissue-level heterogeneity in a biologically plausible target can ultimately contribute to multidimensional disease stratification. The current study establishes that such heterogeneity exists and is clinically associated with response and kidney outcomes, while also showing why tissue staining alone is insufficient to distinguish causal pathway activation from chronic injury, disease severity, or epiphenomenal target expression.

### Strengths and limitations

Strengths of this study include the systematic evaluation of archived human FSGS biopsy tissue in 103 patients, at least 24 months of follow-up for every included patient, explicit operational definitions of TRPC5 and Palladin staining patterns, joint evaluation by two renal pathologists blinded to clinical outcome data, and integration of tissue phenotypes with clinical response and kidney outcomes. The analysis also examined the robustness of the observed associations through treatment-adjusted, IFTA, global-glomerulosclerosis, native-kidney, follow-up-duration, interaction, and incremental-value analyses. Importantly, the revised analytic framework distinguishes observed immunohistochemical phenotype from functional channel activity, causal mechanism, treatment predictiveness, and independent prognostic value.

Several limitations require consideration. First, FSGS etiology could not be uniformly reconstructed because genetic testing, electron-microscopy findings, nephrotic-syndrome adjudication, and systematic evaluation of secondary causes were not consistently available across the historical cohort. The native-kidney sensitivity analysis reduced the potential influence of allograft biopsies but cannot be interpreted as an analysis restricted to primary FSGS. Second, 79 of 182 screened patients were excluded because of insufficient evaluable glomerular tissue or unavailable 24-month clinical outcome data; because complete baseline analytic data were not retained for the excluded group, the magnitude and direction of potential selection bias cannot be quantified. Third, treatment was nonrandomized and heterogeneous, leaving substantial potential for confounding by indication despite treatment-category adjustment. Fourth, IFTA grading was unavailable in 33 patients; the attenuation and wide confidence intervals in the complete-case models limit conclusions regarding independence from chronic structural injury. Exact kidney-failure event dates were also not uniformly available, precluding time-to-event analysis.

The immunohistochemical approach introduces additional limitations. Staining was semiquantitative, and a separate reagent-negative/isotype-matched control and an independent orthogonal target-validation assay were not performed in the original archival staining series. Podocyte-specific colocalization was also not performed. PA5-67556 was used at the supplier-tested dilution for human FFPE immunohistochemistry, and the original staining series included a positive-control tissue together with predefined reproducible glomerular staining criteria; these features support technical assay performance but do not independently establish molecular target identity within human kidney tissue. Although two renal pathologists reached the final classifications by consensus while blinded to clinical outcomes, independent pre-consensus scores were not retained and interobserver agreement could therefore not be quantified retrospectively. Accordingly, the observed immunoreactivity should be interpreted as an operational glomerular TRPC5-directed immunohistochemical phenotype and cannot establish quantitative TRPC5 protein abundance, definitive podocyte-specific localization, membrane-channel activity, calcium flux, Rac1 signaling, or therapeutic dependence. Similarly, Palladin scoring characterized glomerular staining distribution rather than quantitative protein abundance. Finally, the retrospective single-center design and modest number of kidney-failure events limit statistical precision and external generalizability.

The next translational step is therefore not simply a larger retrospective validation of staining–outcome associations, but a prospective, biomarker-integrated study in etiologically characterized FSGS. Such a study should combine standardized treatment exposure with quantitative or digital TRPC5 tissue phenotyping, cell-specific localization, orthogonal protein validation, and functional readouts of the relevant signaling pathway, while integrating genetics, clinical phenotype, and longitudinal proteinuria and kidney-function outcomes. This design would allow determination of whether TRPC5 tissue heterogeneity primarily reflects chronic structural injury, marks a distinct biological state, or identifies a subgroup in which target and pathway activity are sufficiently concordant to have therapeutic relevance.

## Conclusion

PRIME-PATH I demonstrates marked interindividual heterogeneity in glomerular TRPC5 capillary-granular immunoreactivity within morphologically defined FSGS. To our knowledge, this is the first full-length systematic immunohistochemical characterization of TRPC5 across a human FSGS biopsy cohort of this scale, with parallel assessment of Palladin staining distribution. TRPC5 immunoreactivity was associated with 24-month treatment response and kidney failure during follow-up; however, persistence of these associations after adjustment for global glomerulosclerosis, together with attenuation in the IFTA complete-case analysis, underscores chronic structural injury as an important alternative explanation and leaves the independent prognostic value of TRPC5 uncertain. Palladin distribution did not provide significant incremental information beyond TRPC5 and clinical covariates.

By demonstrating previously unrecognized tissue-level heterogeneity in glomerular TRPC5-directed immunoreactivity across a large human FSGS biopsy cohort, PRIME-PATH I defines a distinct precision-pathology dimension linking experimental TRPC5 biology with the evolving precision-therapeutic landscape of podocytopathies. Although they do not establish functional TRPC5 activation or treatment predictiveness, they provide a framework for prospective biomarker-integrated studies incorporating etiologic classification, cell-specific localization, functional pathway assessment, and treatment-response evaluation.

## Data Availability

The datasets presented in this article are not readily available because they contain patient-level retrospective clinical and pathology data subject to patient confidentiality and institutional and ethics restrictions. Requests to access the datasets should be directed to the corresponding author, Zeynep Ural (zeynepkaracan14@gmail.com), and will be considered subject to applicable institutional and ethics requirements.

## References

[1] Rovin BH Adler SG Barratt J Bridoux F Burdge KA Chan TM. KDIGO 2021 clinical practice guideline for the management of glomerular diseases. Kidney Int. (2021) 100(4S):S1–S276. 10.1016/j.kint.2021.05.021 34556256

[2] De Vriese AS Sethi S Nath KA Glassock RJ Fervenza FC. Differentiating primary, genetic, and secondary FSGS in adults: a clinicopathologic approach. J Am Soc Nephrol. (2018) 29(3):759–74. 10.1681/ASN.2017090958 29321142 PMC5827609

[3] Bonilla M Efe O Selvaskandan H Lerma EV Wiegley N. A review of focal segmental glomerulosclerosis classification with a focus on genetic associations. Kidney Med. (2024) 6(6):100826. 10.1016/j.xkme.2024.100826 38765809 PMC11099322

[4] Trachtman H Eddy S Kretzler M. Current and future therapeutics for focal segmental glomerular sclerosis in the era of precision medicine: a review. Am J Kidney Dis. (2026) 87(4):573–81. 10.1053/j.ajkd.2025.10.013 41429299

[5] Altintas MM Agarwal S Sudhini Y Zhu K Wei C Reiser J. Pathogenesis of focal segmental glomerulosclerosis and related disorders. Annu Rev Pathol. (2025) 20:329–53. 10.1146/annurev-pathol-051220-092001 39854184 PMC11875227

[6] D'Agati VD Kaskel FJ Falk RJ. Focal segmental glomerulosclerosis. N Engl J Med. (2011) 365(25):2398–411. 10.1056/NEJMra1106556 22187987

[7] Romagnani P Tang SCW Weins A Huber TB Osafo C Anders HJ. Podocytopathies. Nat Rev Dis Primers. (2025) 11:87. 10.1038/s41572-025-00671-w 41381622

[8] Meliambro K He JC Campbell KN. Podocyte-targeted therapies—progress and future directions. Nat Rev Nephrol. (2024) 20(10):643–58. 10.1038/s41581-024-00843-z 38724717

[9] Tian X Ishibe S. Targeting the podocyte cytoskeleton: from pathogenesis to therapy in proteinuric kidney disease. Nephrol Dial Transplant. (2016) 31(10):1577–83. 10.1093/ndt/gfw021 26968197 PMC5039341

[10] Hengel FE Dehde S Lassé M Zahner G Seifert L Schnarre A. Autoantibodies targeting nephrin in podocytopathies. N Engl J Med. (2024) 391(5):422–33. 10.1056/NEJMoa2314471 38804512

[11] Raglianti V Angelotti ML Cirillo L Ravaglia F Landini S Palazzo V. Anti-slit diaphragm antibodies on kidney biopsy identify pediatric patients with steroid-resistant nephrotic syndrome responsive to second-line immunosuppressants. Kidney Int. (2024) 106(6):1124–34. 10.1016/j.kint.2024.09.006 39368741

[12] Weins A Batal I Romagnani P Singh G Raj R Andeen N. What’s new in medical renal pathology 2025: updates on podocytopathy and immunofluorescence staining in medical kidney. J Pathol Transl Med. (2025) 59(4):269–72. 10.4132/jptm.2025.06.19 40665660 PMC12264474

[13] Haydak J Azeloglu EU. Role of biophysics and mechanobiology in podocyte physiology. Nat Rev Nephrol. (2024) 20(6):371–85. 10.1038/s41581-024-00815-3 38443711 PMC12103212

[14] Tian D Jacobo SMP Billing D Rozkalne A Gage SD Anagnostou T. Antagonistic regulation of actin dynamics and cell motility by TRPC5 and TRPC6 channels. Sci Signal. (2010) 3(145):ra77. 10.1126/scisignal.2001200 20978238 PMC3071756

[15] Schaldecker T Kim S Tarabanis C Tian D Hakroush S Castonguay P. Inhibition of the TRPC5 ion channel protects the kidney filter. J Clin Invest. (2013) 123(12):5298–309. 10.1172/JCI71165 24231357 PMC3859394

[16] Zhou Y Castonguay P Sidhom E-H Clark AR Dvela-Levitt M Kim S. A small-molecule inhibitor of TRPC5 ion channels suppresses progressive kidney disease in animal models. Science. (2017) 358(6368):1332–6. 10.1126/science.aal4178 29217578 PMC6014699

[17] Wang X Dande RR Yu H Samelko B Miller RE Altintas MM. TRPC5 does not cause or aggravate glomerular disease. J Am Soc Nephrol. (2018) 29(2):409–15. 10.1681/ASN.2017060682 29061651 PMC5791075

[18] Polat OK Isaeva E Sudhini YR Knott B Zhu K Noben M. The small GTPase regulatory protein Rac1 drives podocyte injury independent of cationic channel protein TRPC5. Kidney Int. (2023) 103(6):1056–62. 10.1016/j.kint.2023.01.016 36750145 PMC10200725

[19] Staruschenko A Alexander RT Caplan MJ Ilatovskaya DV. Calcium signalling and transport in the kidney. Nat Rev Nephrol. (2024) 20(8):541–55. 10.1038/s41581-024-00835-z 38641658 PMC12036682

[20] Walsh L Reilly JF Cornwall C Gaich GA Gipson DS Heerspink HJL. Safety and efficacy of GFB-887, a TRPC5 channel inhibitor, in patients with focal segmental glomerulosclerosis, treatment-resistant minimal change disease, or diabetic nephropathy: tRACTION-2 trial design. Kidney Int Rep. (2021) 6(10):2575–84. 10.1016/j.ekir.2021.07.006 34622097 PMC8484122

[21] Staruschenko A Ma R Palygin O Dryer SE. Ion channels and channelopathies in glomeruli. Physiol Rev. (2023) 103(1):787–854. 10.1152/physrev.00013.2022 36007181 PMC9662803

[22] Endlich N Schordan E Cohen CD Kretzler M Lewko B Welsch T. Palladin is a dynamic actin-associated protein in podocytes. Kidney Int. (2009) 75(2):214–26. 10.1038/ki.2008.486 19116644

[23] Artelt N Ludwig TA Rogge H Kavvadas P Siegerist F Blumenthal A. The role of palladin in podocytes. J Am Soc Nephrol. (2018) 29(6):1662–78. 10.1681/ASN.2017091039 29720549 PMC6054350

[24] Chang EH Gasim AH Kerber ML Patel JB Glaubiger SA Falk RJ. Palladin is upregulated in kidney disease and contributes to epithelial cell migration after injury. Sci Rep. (2015) 5:7695. 10.1038/srep07695 25573828 PMC4648347

[25] Yamamoto N Sakai N Yamamura Y Kaikoi D Hayashi D Matsuno T. Fibroblast-specific palladin drives kidney fibrosis via MRTF–SRF signaling. J Pathol. (2026) 268(1):40–53. 10.1002/path.6485 41131992 PMC12699245

[26] Yamamoto N Sakai N Kaikoi D Matsuno T Koshino A Sako K. Interstitial palladin expression is associated with increased risk of end-stage kidney disease and death in patients with biopsy-proven diabetic kidney disease. Clin Exp Nephrol. (2026) 30(6):875–82. 10.1007/s10157-026-02852-7 41886153

[27] Troost JP Trachtman H Spino C Kaskel FJ Friedman A Moxey-Mims MM. Proteinuria reduction and kidney survival in focal segmental glomerulosclerosis. Am J Kidney Dis. (2021) 77(2):216–25. 10.1053/j.ajkd.2020.04.014 32791086 PMC7854818

[28] Troyanov S Wall CA Miller JA Scholey JW Cattran DC. Focal and segmental glomerulosclerosis: definition and relevance of a partial remission. J Am Soc Nephrol. (2005) 16(4):1061–8. 10.1681/ASN.2004070593 15716334

[29] Reiser J Polu KR Möller CC Kenlan P Altintas MM Wei C. TRPC6 is a glomerular slit diaphragm-associated channel required for normal renal function. Nat Genet. (2005) 37(7):739–44. 10.1038/ng1592 15924139 PMC1360984

[30] Winn MP Conlon PJ Lynn KL Farrington MK Creazzo T Hawkins AF. A mutation in the TRPC6 cation channel causes familial focal segmental glomerulosclerosis. Science. (2005) 308(5729):1801–4. 10.1126/science.1106215 15879175

[31] Möller CC Wei C Altintas MM Li J Greka A Ohse T. Induction of TRPC6 channel in acquired forms of proteinuric kidney disease. J Am Soc Nephrol. (2007) 18(1):29–36. 10.1681/ASN.2006091010 17167110

[32] Kistler AD Singh G Altintas MM Yu H Fernandez IC Gu C. Transient receptor potential channel 6 (TRPC6) protects podocytes during complement-mediated glomerular disease. J Biol Chem. (2013) 288(51):36598–609. 10.1074/jbc.M113.488122 24194522 PMC3868772

[33] Riehle M Büscher AK Gohlke B-O Kaßmann M Kolatsi-Joannou M Bräsen JH. TRPC6 G757D loss-of-function mutation associates with FSGS. J Am Soc Nephrol. (2016) 27(9):2771–83. 10.1681/ASN.2015030318 26892346 PMC5004639

[34] Trachtman H Kretzler M Gesualdo L Cross N Workeneh B Kaufeld J. TRPC6 inhibition for the treatment of focal segmental glomerulosclerosis: a randomised, placebo-controlled, phase 2 trial of BI 764198. Lancet. (2026) 407(10528):587–98. 10.1016/S0140-6736(25)02255-X 41616795

[35] Xie Y Liu F. Precision medicine for focal segmental glomerulosclerosis. Kidney Res Clin Pract. (2024) 43(6):709–23. 10.23876/j.krcp.23.227 38325863 PMC11615440

[36] Zheng Q Gu X He JC Xie J. Progress in therapeutic targets on podocyte for alport syndrome. J Transl Int Med. (2024) 12(2):129–33. 10.2478/jtim-2024-0005 38812923 PMC11135632

[37] Zhu Y Xu G. Advances in focal segmental glomerulosclerosis treatment from the perspective of the newest mechanisms of podocyte injury. Drug Des Devel Ther. (2025) 19:857–75. 10.2147/DDDT.S498457 39935575 PMC11812565

