# Supplementary material for: Glomerular TRPC5 and Palladin immunohistochemical profiling in focal segmental glomerulosclerosis: tissue heterogeneity and exploratory clinical associations (PRIME-PATH I)
Source: Front Med (Lausanne). 2026 Oct 22;13:1924319. doi: 10.3389/fmed.2026.1924319 (PMC13735589; doi:10.3389/fmed.2026.1924319)
Supplement: Supplementary file 1 [file Datasheet1.pdf]

Supplementary Material

Glomerular TRPC5 and Palladin Immunohistochemical Profiling in Focal Segmental Glomerulosclerosis: Tissue Heterogeneity and Exploratory Clinical Associations (PRIME-PATH I)

Supplementary Table S1. Sensitivity, incremental-value, and interaction analyses

Analysis	Estimate/result	Statistical test	Notes
Follow-up duration by TRPC5 status	TRPC5−: 44 (36–72) months; TRPC5+: 48 (37–60) months	Mann–Whitney U, P=0.763	No evidence of differential follow-up duration between TRPC5 groups
Native-kidney sensitivity analysis: poor response	TRPC5−: 9/31 (29.0%); TRPC5+: 38/63 (60.3%)	Fisher's exact, P=0.008	Association persisted after exclusion of allograft biopsies; this analysis does not constitute etiologic classification of primary FSGS
Native-kidney sensitivity analysis: kidney failure	TRPC5−: 3/31 (9.7%); TRPC5+: 24/63 (38.1%)	Fisher's exact, P=0.004	Association persisted after exclusion of allograft biopsies; this analysis does not constitute etiologic classification of primary FSGS
IFTA complete-case sensitivity analysis: poor response	TRPC5: OR 2.27 (95% CI, 0.52–9.97); IFTA grade: OR 2.73 (95% CI, 1.11–6.71)	TRPC5 P=0.279; IFTA P=0.029	n=70; TRPC5 estimate was attenuated after adjustment for chronic structural injury
IFTA complete-case sensitivity analysis: kidney failure	TRPC5: OR 4.12 (95% CI, 0.55–31.12); IFTA grade: OR 2.55 (95% CI, 0.90–7.22)	TRPC5 P=0.170; IFTA P=0.078	n=70; estimates were imprecise
Global-sclerosis sensitivity analysis: poor response	TRPC5: OR 3.42 (95% CI, 1.15–10.20); global sclerosis per 10% increase: OR 1.32 (95% CI, 1.03–1.68)	TRPC5 P=0.027; global sclerosis P=0.028	Full cohort (n=103); percentage of globally sclerotic glomeruli added to the primary model
Global-sclerosis sensitivity analysis: kidney failure	TRPC5: OR 5.54 (95% CI, 1.25–24.63); global sclerosis per 10% increase: OR 1.12 (95% CI, 0.86–1.47)	TRPC5 P=0.024; global sclerosis P=0.392	Full cohort (n=103); percentage of globally sclerotic glomeruli added to the primary model
Incremental value of Palladin distribution: poor response	AIC 112.32 without Palladin vs 114.07 with Palladin; apparent AUC 0.850 vs 0.849	Likelihood-ratio P=0.620	No evidence that adding diffuse vs discontinuous Palladin distribution improved adjusted model fit
TRPC5 × Palladin-distribution interaction: poor response	OR 0.28 (95% CI, 0.018–4.36)	Interaction P=0.364; nested likelihood-ratio P=0.342	No evidence of interaction; confidence interval was wide
Incremental value of Palladin distribution: kidney failure	AIC 100.32 without Palladin vs 100.46 with Palladin; apparent AUC 0.853 vs 0.861	Likelihood-ratio P=0.173	No significant improvement in adjusted model fit after addition of Palladin distribution
TRPC5 × Palladin-distribution interaction: kidney failure	Not stably estimable	—	Zero kidney-failure events in the TRPC5−/Palladin-discontinuous group and sparse subgroup data produced separation

The primary adjusted model included TRPC5 status, Palladin distribution, age, sex, baseline eGFR, and log2-transformed baseline 24-hour urinary protein excretion. IFTA and global-sclerosis analyses additionally incorporated the corresponding chronicity measure. All 103 included patients had at least 24 months of follow-up. Palladin analyses compare diffuse versus discontinuous glomerular capillary-granular distribution; there was no Palladin-negative analytic group. AIC, Akaike information criterion; AUC, area under the receiver-operating-characteristic curve; CI, confidence interval; eGFR, estimated glomerular filtration rate; FSGS, focal segmental glomerulosclerosis; IFTA, interstitial fibrosis and tubular atrophy; OR, odds ratio; TRPC5, transient receptor potential canonical 5.
